# Clinical Predictors of Nontuberculous Mycobacteria Lung Disease and Coisolates of Potential Pathogenic Microorganisms in Noncystic Fibrosis Bronchiectasis

**DOI:** 10.1093/ofid/ofae427

**Published:** 2024-07-18

**Authors:** Ping-Huai Wang, Chin-Chung Shu, Chau-Chyun Sheu, Chia-Ling Chang, Meng-Heng Hsieh, Wu-Huei Hsu, Ming-Tsung Chen, Wei-Fan Ou, Yu-Feng Wei, Tsung-Ming Yang, Chou-Chin Lan, Cheng-Yi Wang, Chih-Bin Lin, Ming-Shian Lin, Yao-Tung Wang, Ching-Hsiung Lin, Shih-Feng Liu, Meng-Hsuan Cheng, Yen-Fu Chen, Wen-Chien Cheng, Chung-Kan Peng, Ming-Cheng Chan, Ching-Yi Chen, Lun-Yu Jao, Ya-Hui Wang, Chi-Jui Chen, Shih-Pin Chen, Yi-Hsuan Tsai, Shih-Lung Cheng, Horng-Chyuan Lin, Jung-Yien Chien, Hao-Chien Wang

**Affiliations:** Division of Thoracic Medicine, Far Eastern Memorial Hospital, New Taipei City, Taiwan; School of Medicine, National Yang Ming Chiao Tung University, Taipei, Taiwan; Department of Internal Medicine, National Taiwan University Hospital, Taipei, Taiwan; College of Medicine, National Taiwan University, Taipei, Taiwan; Division of Pulmonary and Critical Care Medicine, Department of Internal Medicine, Kaohsiung Medical University Hospital, Kaohsiung Medical University, Kaohsiung, Taiwan; Department of Internal Medicine, School of Medicine, College of Medicine, Kaohsiung Medical University, Kaohsiung, Taiwan; Department of Internal Medicine, National Taiwan University Hospital Hsin-Chu Branch, Hsin-Chu, Taiwan; Graduate Institute of Clinical Medicine, College of Medicine, National Taiwan University, Taipei, Taiwan; Department of Thoracic Medicine, Chang Gung Memorial Hospital at Linkou, Taoyuan City, Taiwan; College of Medicine, Chang Gung University, Taoyuan, Taiwan; Division of Pulmonary and Critical Care Medicine, Department of Internal Medicine, China Medical University Hospital, Taichung City, Taiwan; Critical Medical Center, China Medical University Hospital, Taichung, Taiwan; Division of Pulmonary and Critical Care Medicine, Department of Internal Medicine, Tri-Service General Hospital, National Defense Medical Center, Taipei, Taiwan; Division of Chest Medicine, Department of Internal Medicine, Taichung Veterans General Hospital, Taichung City, Taiwan; Department of Internal Medicine, E-Da Cancer Hospital, I-Shou University, Kaohsiung, Taiwan; School of Medicine for International Students, College of Medicine, I-Shou University, Kaohsiung, Taiwan; Division of Pulmonary and Critical Care Medicine, Chiayi Chang Gung Memorial Hospital, Chiayi, Taiwan; Division of Pulmonary Medicine, Department of Internal Medicine, Taipei Tzu Chi Hospital, Buddhist Tzu Chi Medical Foundation, New Taipei City, Taiwan, Republic of China; Department of Internal Medicine, Cardinal Tien Hospital and School of Medicine, College of Medicine, Fu Jen Catholic University, New Taipei City, Taiwan; Division of Pulmonary Medicine, Department of Internal Medicine, Hualien Tzu Chi Hospital, Buddhist Tzu Chi Medical Foundation, Hualien, Taiwan; School of Medicine, Tzu-Chi University, Hualien, Taiwan; Department of Respiratory Care, Chang Gung University of Science and Technology, Chiayi City, Taiwan; Division of Pulmonary Medicine, Department of Internal Medicine, Chung Shan Medical University Hospital, Taichung, Taiwan; School of Medicine, Chung Shan Medical University, Taichung, Taiwan; Department of Internal Medicine, Division of Chest Medicine, Changhua Christian Hospital, Changhua, Taiwan; Institute of Genomics and Bioinformatics, National Chung Hsing University, Taichung, Taiwan; PhD Program in Translational Medicine, National Chung Hsing University, Taichung, Taiwan; Department of Recreation and Holistic Wellness, MingDao University, Changhua, Taiwan; Division of Pulmonary & Critical Care Medicine, Department of Internal Medicine, Kaohsiung Chang Gung Memorial Hospital, Kaohsiung City, Taiwan; Department of Respiratory Therapy, Kaohsiung Chang Gung Memorial Hospital, Kaohsiung City, Taiwan; College of Medicine, Chang Gung University, Taoyuan, Taiwan; Division of Pulmonary and Critical Care Medicine, Department of Internal Medicine, Kaohsiung Medical University Hospital, Kaohsiung Medical University, Kaohsiung, Taiwan; Department of Respiratory Therapy, College of Medicine, Kaohsiung Medical University, Kaohsiung, Taiwan; Department of Internal Medicine, National Taiwan University Hospital Yun-Lin branch, Yun-Lin, Taiwan; Thoracic Medicine Center, Department of Medicine and Surgery, National Taiwan University Hospital Yun-Lin branch, Yun-Lin, Taiwan; Division of Pulmonary and Critical Care Medicine, Department of Internal Medicine, China Medical University Hospital, Taichung City, Taiwan; Division of Pulmonary and Critical Care Medicine, Department of Internal Medicine, Tri-Service General Hospital, National Defense Medical Center, Taipei, Taiwan; Department of Medical Planning, Medical Affairs Bureau Ministry of National Defense, Taipei City, Taiwan; Department of Critical Care Medicine, Taichung Veterans General Hospital, Taichung City, Taiwan; School of Post Baccalaureate Medicine, College of Medicine National Chung Hsing University, Taichung City, Taiwan; Department of Internal Medicine, E-Da Hospital, I-Shou University, Kaohsiung, Taiwan; School of Medicine, Tzu-Chi University, Hualien, Taiwan, Republic of China; Medical Research Center, Cardinal Tien Hospital and School of Medicine, College of Medicine, Fu Jen Catholic University, New Taipei City, Taiwan; Division of Pulmonary Medicine, Department of Internal Medicine, Hualien Tzu Chi Hospital, Buddhist Tzu Chi Medical Foundation, Hualien, Taiwan; Division of Pulmonary Medicine, Department of Internal Medicine, Chung Shan Medical University Hospital, Taichung, Taiwan; School of Medicine, Chung Shan Medical University, Taichung, Taiwan; Division of Pulmonary & Critical Care Medicine, Department of Internal Medicine, Kaohsiung Chang Gung Memorial Hospital, Kaohsiung City, Taiwan; Department of Pulmonary Medicine, Lee's Clinic, Pingtung, Taiwan; Division of Thoracic Medicine, Far Eastern Memorial Hospital, New Taipei City, Taiwan; Department of Chemical Engineering and Materials Science, Yuan-Ze University, Taoyuan City, Taiwan; Department of Thoracic Medicine, Chang Gung Memorial Hospital at Linkou, Taoyuan City, Taiwan; College of Medicine, Chang Gung University, Taoyuan, Taiwan; Department of Respiratory Therapy, Chang Gung Memorial Hospital at Linkou, Taoyuan City, Taiwan; Department of Internal Medicine, National Taiwan University Hospital, Taipei, Taiwan; College of Medicine, National Taiwan University, Taipei, Taiwan; College of Medicine, National Taiwan University, Taipei, Taiwan; Department of Medicine National Taiwan University Cancer Center, National Taiwan University College of Medicine, Taipei, Taiwan

**Keywords:** bronchiectasis, nontuberculous mycobacteria, potential pathogenic microorganisms, risk factors, outcome

## Abstract

**Background:**

In bronchiectasis, nontuberculous mycobacteria (NTM) lung disease (NTM-LD) is a well-known coexisting infection. However, microorganism coisolates and clinical NTM-LD predictors are poorly studied.

**Methods:**

Patients with bronchiectasis diagnosed by means of computed tomography between January 2017 and June 2020 were screened, using the date of computed tomography as the index date. Those with a major bronchiectasis diagnosis in ≥2 follow-up visits after the index date were enrolled in the study, and NTM-LD occurrence and its association with pneumonia and hospitalization within 1 year were analyzed.

**Results:**

Of the 2717 participants, 79 (2.9%) had NTM-LD diagnosed. The factors associated with NTM-LD included hemoptysis, postinfectious bronchiectasis, a tree-in-bud score ≥2, a modified Reiff score ≥4, and chronic obstructive pulmonary disease (adjusted odds ratios, 1.80, 2.36, 1.78, 2.95, and 0.51, respectively). Compared with patients in the non-NTM group, those with NTM-LD had higher rates of hospitalization (15.9% vs 32.9%; *P* < .001) and pneumonia (9.8% vs 20.3%; *P* = .003). *Pseudomonas aeruginosa* was the most common microorganism in those with NTM-LD and those in the non-NTM group (10.1% vs 7.8%; *P* = .40). However, compared with those in the non-NTM group, *Acinetobacter baumannii* and *Escherichia coli* were more prevalent in patients with NTM-LD (0.7% vs 3.8% [*P* = .03%] and 1.0% vs 3.8% [*P* = .05], respectively).

**Conclusions:**

Postinfectious bronchiectasis with hemoptysis, higher radiological involvement, and a tree-in-bud pattern were associated with NTM-LD risk. The rate of *A baumannii* and *E coli* coisolation was higher in bronchiectasis populations with NTM-LD.

## Teaser

Factors associated with nontuberculous mycobacteria lung disease (NTM-LD) included hemoptysis, postinfectious bronchiectasis, a greater radiological extent of bronchiectasis, and tree-in-bud. *Acinetobacter baumannii* and *Escherichia coli* were more prevalent in the NTM-LD than in the non-NTM group.

Noncystic fibrosis bronchiectasis is reported to have a prevalence of 4–5 cases per 1000 people [1, 2]. Because its pathophysiology is associated with impaired local immune defense, individuals with bronchiectasis are susceptible to infection, which can induce excessive inflammation driven mainly by neutrophils, causing further bronchial wall damage [3]. This leads to a vicious cycle that worsens bronchiectasis progression. Because bacterial infections can significantly affect bronchiectasis severity, chronic *Pseudomonas aeruginosa* isolation is part of bronchiectasis severity scoring systems, such as the bronchiectasis severity index and FACED [4, 5].

Because nodular bronchiectasis is a major radiological pattern in nontuberculous mycobacteria (NTM) lung disease (NTM-LD) [6], it is sometimes associated with NTM, although it might be a consequence of NTM-LD [7]. A 2022 meta-analysis reported that in bronchiectasis, NTM-LD has a prevalence of 10% [8]. Although some studies indicate that NTM-LD might increase mortality and hospitalization rates [9, 10], reports are inconsistent. Although extended-duration macrolide use is recommended for bronchiectasis with frequent exacerbations [11], preventing bronchiectasis exacerbation using macrolide monotherapy might lead to macrolide resistance in simultaneously occult NTM-LD [12]. To avoid such pitfalls, high clinical suspicion and adequate microbiological surveys are crucial.

Because the clinical manifestations of bronchiectasis and NTM-LD are similar, it is important to identify NTM-LD risk factors in patients with bronchiectasis for early diagnosis. Several registry studies in the United States and Europe have attempted to investigate the status in their regions [13–16]. However, geographic factors influence the epidemiology, microbiology, and clinical features of NTM-LD in patients with bronchiectasis. In addition, bacterial infection is a common bronchiectasis complication. To address these issues, we retrospectively assessed NTM-LD characteristics in patients with bronchiectasis in Taiwan to identify the predictors and outcomes of bronchiectasis, as well as to investigate bacterial coisolation in patients with concomitant NTM-LD and bronchiectasis.

## METHODS

### Participant Enrollment

This retrospective case-control study was conducted at 16 sites located throughout Taiwan. The chest computed tomography (CT) reports from January 2017 to June 2020 were screened. Patients aged >20 years, whose CT reports mentioned typical bronchiectasis findings [12], were considered as study candidates, with CT dates serving as the index dates. Patients who had ≥2 outpatient clinic follow-up visits and had bronchiectasis diagnosed after the index date without human immunodeficiency virus infection or cystic fibrosis were included in the study. The study received ethical approval from the Research Ethics Review Committee of Far Eastern Memorial Hospital (FEMH-111015-E) and the institutional review boards of the other sites. Informed consent was waived because the study is retrospective.

### Clinical and Demographic Data Acquisition

Demographic data were screened within 1 year before or after the index date. Data on symptoms, lung function, blood tests, microbiological examinations, and antibiotic use lasting >3 months were obtained from medical records taken within 1 year of the index date. The demographic and clinical data collected closest to the index date were selected. In addition, data on comorbid conditions were obtained from the medical history recorded before the index date. Heart failure, coronary artery disease, and arrhythmia denoted cardiovascular disease. An estimated glomerular filtration rate of <45 mL/min/1.73 m^2^ indicated chronic kidney disease. Postinfectious bronchiectasis was considered in case of a history of pneumonia or tuberculosis before bronchiectasis diagnosis. Results of microbiological or serological analyses, including serum antibody testing, galactomannan test, cultures, and polymerase chain reaction testing of respiratory specimens, were used to confirm *Aspergillus* isolation. NTM-LD diagnosis was based on the 2020 American Thoracic Society/European Respiratory Society/European Society of Clinical Microbiology and Infectious Diseases/Infectious Diseases Society of America microbiological guidelines [17]. Specifically, it required the isolation of the same NTM species in ≥2 positive sets of separately and consecutively expectorated sputum cultures or 1 set of bronchial lavage specimens.

### CT Interpretation

CT scans were interpreted by a pulmonologist and a radiologist, and any discrepancies were resolved through discussion. Modified Reiff scoring was used to determine bronchiectasis severity [4], which assessed each lobe's involvement and its degree of bronchial dilation. Each lung has 3 lobes and the lingular segment was considered a separate lobe of the left lung. Bronchial dilation was scored 1, 2, or 3, indicating tubular, varicose, and cystic, respectively, with the score’s sum of the 6 lobes giving the modified Reiff score. The presence of centrilobular nodules in the bronchioles and their branching was indicated by tree-in-bud (TIB) patterns on CT images [18], and the TIB pattern in each lobe was given a score of 1, for a maximum score of 6 for the 6 lobes.

### Outcomes

Hospitalization and pneumonia (indicated by respiratory symptom changes, sputum characteristics, and chest radiological findings requiring additional antibiotic treatment) data were retrieved from the medical data recorded within 1 year of the index date. Hospitalization data were retrieved in cases of primary pneumonia or bronchiectasis diagnosis on admission with exacerbation. Data on mortality outcomes until June 2022 were obtained from medical records.

### Statistical Analyses

For continuous and categorical variables, data are presented as means with standard deviation (SD) and numbers with percentages, respectively. Statistical analyses were done using SPSS software, version 19 (IBM). For continuous and categorical variables, differences were compared using Student *t* and a χ^2^ tests, respectively. The Youden index was used to determine the modified Reiff and TIB score cutoff points. After univariable analysis, variables with *P* values <.1 were included in the multivariable analysis, in which the forward input method was used for the logistic regression model. Kaplan-Meier analysis was used to estimate the 3-year mortality rate. Hazard ratios (HRs) comparing 3-year mortality rates by NTM-LD and NTM-LD treatment status were estimated by Cox proportional hazard model. Differences were considered statistically significant at *P* < .05.

## RESULTS

### Participant Enrollment and Grouping

Of the 2751 participants enrolled in the study, 34 were excluded, who had active tuberculosis diagnosed within 1 year of follow-up after the index date (Supplementary Figure 1). Of the remaining 2717 participants, 2535 whose respiratory specimens did not have positive mycobacteria cultures formed the non-NTM group, and 79 (2.9%; incidence rate, 29 cases per 1000 person-years) who met the NTM-LD diagnosis criteria, formed the NTM-LD group. The remaining 103 participants (3.7%) had only 1 set of positive NTM cultures or 2 different NTM isolate species in the year after the index date.

### NTM-LD Species Distribution

**Figure 1.**
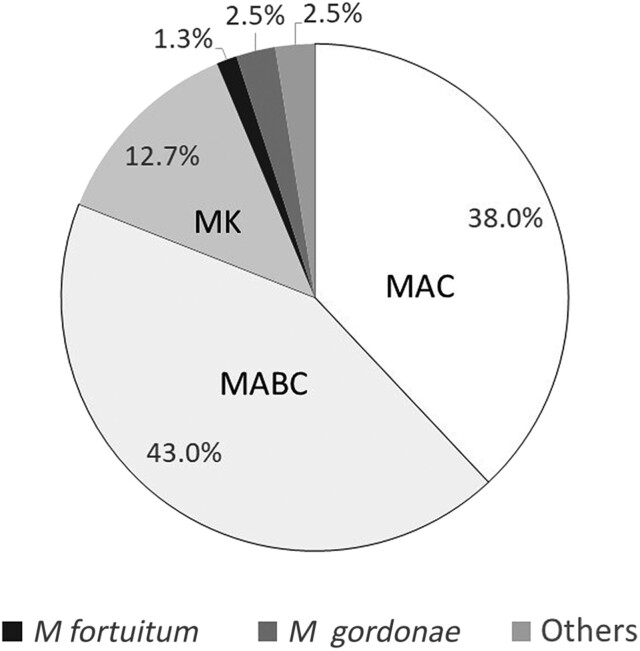
Percentage of nontuberculous mycobacteria (NTM) species in individuals with NTM–lung disease (LD) associated with bronchiectasis. *Mycobacterium abscessus complex* (MABC) accounted for 43% of NTM-LD cases, followed by *Mycobacterium avium complex* (MAC) (38%), *Mycobacterium kansasii* (MK) (12.7%), *Mycobacterium gordonae* (2.5%), and *Mycobacterium fortuitum* (1.3%).

The most common pathogens in the NTM-LD group were *Mycobacterium abscessus* complex (MABC) (34 of 79 [43%]) and *Mycobacterium avium* complex (MAC) (30 of 79 [38%]), while *Mycobacterium kansasii* (10 of 79 [12.7%]) was the third most common (Figure 1). Regarding NTM colonization, MAC (28 of 103 [27.2%]) was the most common, followed by MABC (17 of 103 [16.5%]) (Supplementary Table 1).

### Demographic and comorbidity Characteristics

There were no significant differences in sex and age between NTM-LD and non-NTM groups (Table 1). The body mass index (BMI) was lower in the NTM-LD than in the non-NTM group (BMI, 20.4 ± 3.2 vs 22.1 ± 4.0 [calculated as weight in kilograms divided by height in meters squared]; *P* < .001). Hemoptysis and phlegm were more frequent in the NTM-LD than in the non-NTM group (39.2% vs 21.9% [*P* = .001] and 84.8% vs 72.5% [*P* = .01], respectively). Postinfectious bronchiectasis was more common in the NTM-LD than in the non-NTM group (65.8% vs 42.5%; *P* < .001). However, except for chronic obstructive pulmonary disease (COPD), rates of comorbid conditions were similar in the NTM-LD and non-NTM groups. Strikingly, compared with the non-NTM group, smoking history, and COPD were lower in the NTM-LD group. Lung function tests had been conducted in 1403 participants (53.7%). The proportions of obstructive ventilation defects and forced vital capacity did not differ significantly (Supplementary Table 2). However, the rate of home oxygen use was higher in the NTM-LD than in the non-NTM group (5.1% vs 2.0%; *P* = .083). After anti-NTM treatment exclusion, chronic macrolide use for >3 months did not differ significantly between the non-NTM and NTM-LD groups (10.8% vs 12.7%; *P* = .21) (Supplementary Table 3).

**Table 1.** Demographic and Clinical Characteristics of Study Participants

Characteristic	Participants, No. (%)^a^			*P* Value
	All (n = 2614)	Non-NTM Group (n = 2535)	NTM-LD Group (n = 79)	
Male sex	1090 (41.7)	1064 (42.0)	26 (32.9)	.13
Age, mean (SD), y	66.4 (12.0)	66.5 (12.0)	64.6 (10.8)	.16
BMI, mean (SD)^b^	22.0 (4.0) (n = 2488)	22.1 (4.0) (n = 2413)	20.4 (3.2) (n = 75)	<.001
BMI <18.5^b^	426 (17.1)	405 (16.8)	21 (26.6)	.02
Smoking history (yes/no)	665/1949 (25.4/74.6)	654/1881 (25.8/74.2)	11/68 13.9/86.1	.02
Symptoms				
Hemoptysis	586 (22.4)	555 (21.9)	31 (39.2)	.001
Dyspnea	1078 (41.2)	1050 (41.4)	28	.30
Phlegm	1905 (72.9)	1838 (72.5)	67 (84.8)	.01
Comorbid conditions				
Hypertension	886 (33.9)	866 (34.2)	20 (25.3)	.12
DM	426 (16.3)	417 (16.4)	9 (11.4)	.28
CV disease	896 (34.3)	876 (34.6)	20 (25.3)	.12
CKD	194 (7.4)	191 (7.5)	3 (3.8)	.28
ESRD	39 (1.5)	37 (1.5)	2 (2.5)	.33
Cirrhosis	35 (1.3)	34 (1.3)	1 (1.3)	>.99
Asthma	521 (19.9)	509 (20)	12 (15.2)	.32
COPD	863 (33.0)	846 (33.4)	17 (21.5)	.03
Postinfectious status	1141 (43.6)	1088 (42.9)	52 (65.8)	<.001
Prior pneumonia	917 (35.1)	870 (34.3)	47 (59.5)	<.001
Prior tuberculosis	388 (33.1)	369 (14.6)	19 (21.5)	.02
Cancer	194 (14.8)	191 (7.5)	3 (3.8)	.28
Autoimmunity	68 (2.6)	66 (2.6)	2 (2.5)	>.99
Home oxygen use	55 (2.1)	51 (2.0)	4 (5.1)	.08
TIB score, mean (SD)	1.3 (1.8)	1.3 (1.8)	2.2 (2.2)	<.001
TIB score ≥2	863 (33.0)	821 (32.4)	42 (53.2)	<.001
Modified Reiff score, mean (SD)	4.9 (3.3)	4.9 (3.3)	6.3 (3.2)	<.001
Modified Reiff score ≥4	1527 (58.4)	1462 (57.7)	65 (82.3)	<.001

Abbreviations: BMI, body mass index; CKD, chronic kidney disease; COPD, chronic obstructive pulmonary disease; CV disease, cardiovascular disease; DM, diabetes mellitus; ESRD, end-stage renal disease; LD, lung disease; NTM, nontuberculous mycobacteria; SD, standard deviation; TIB, tree-in-bud.

^a^Data represent no. (%) of study participants unless otherwise specified.

^b^BMI calculated as weight in kilograms divided by height in meters squared.

### Radiological Characteristics

Modified Reiff scores were higher in the NTM-LD than in the non-NTM group (mean score [SD], 6.3 [3.2] vs 4.9 [3.3]; *P* < .001) (Table 1). Furthermore, compared with the non-NTM group, the NTM-LD group had more lobes with TIB patterns (mean TIB score [SD], 2.2 [2.2] vs 1.3 [1.8]; *P* < .001). Based on the Youden index, the modified Reiff and TIB score cutoff points were 4 and 2, respectively, and the proportion of participants with modified Reiff and TIB scores of ≥4 and ≥2, respectively, were higher in the NTM-LD group than in the non-NTM group (82.3% vs 57.7% and 53.2% vs 32.4%, respectively; both *P* < .001).

### Multivariate Analysis of NTM-LD–Associated Factors

Multivariate analysis identified hemoptysis (adjusted odds ratio, 1.80 [95% confidence interval (CI), 1.11–2.92]; *P* = .02), postinfectious bronchiectasis (2.36 [1.49–3.90]; *P* = .001), TIB scores ≥2 (1.78 [1.11–2.86]; *P* = .02), and modified Reiff scores ≥4 (2.95 [1.56–5.57]; *P* = .001) as NTM-LD risk factors (Table 2). However, COPD was identified as a protective factor against NTM-LD (adjusted odds ratio, 0.51 [95% CI, .29–.91]; *P* = .02).

**Table 2.** Factors Associated With Nontuberculous Mycobacteria Lung Disease by Multivariable Analysis

Factor	Crude OR (95% CI)	*P* Value	Adjusted OR (95% CI)	*P* Value
BMI <18.5^a^	1.93 (1.15–3.22)	.02	1.27 (.75–2.15)	.38
Smoking history				
Current or former smoker	Reference	…	…	…
Nonsmoker	0.47 (.25–.89)	.018	0.64 (.31–1.29)	.21
Hemoptysis	2.30 (1.45–3.66)	.001	1.80 (1.11–2.92)	.02
Phlegm	2.12 (1.14–3.94)	.014	1.81 (.91–3.60)	.09
COPD	0.55 (.32–.94)	.029	0.51 (.29–.91)	.02
Postinfectious status	2.56 (1.60–4.10)	<.001	2.36 (1.49–3.90)	.001
TIB score ≥2	2.37 (1.51–3.72)	<.001	1.78 (1.11–2.86)	.02
Modified Reiff score ≥4	3.41 (1.90–6.10)	<.001	2.95 (1.56–5.57)	.001
Home oxygen use	2.60 (.92–7.37)	.080	1.91 (.65–5.67)	.24

Abbreviations: BMI, body mass index; CI, confidence interval; COPD, chronic obstructive pulmonary disease; OR, odds ratio; TIB, tree-in-bud.

^a^BMI calculated as weight in kilograms divided by height in meters squared.

**Figure 2.**
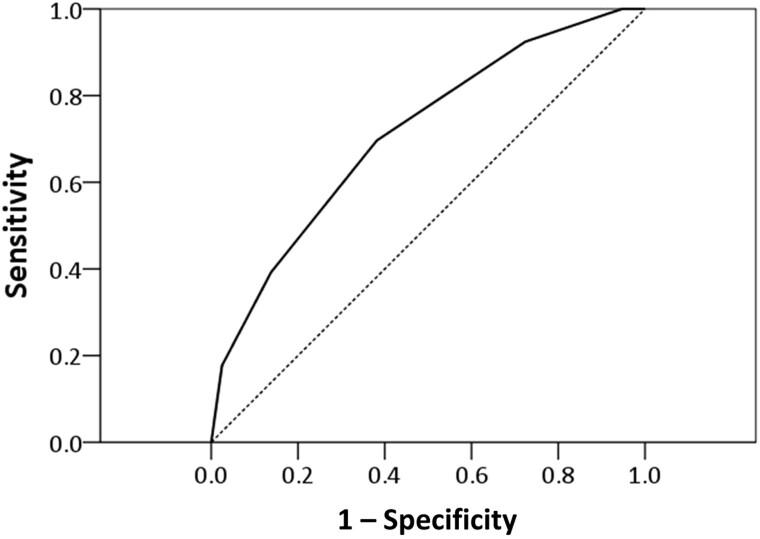
Receiver operating curve of risk factors associated with nontuberculous mycobacteria lung disease (NTM-LD). The risk factors for NTM-LD were identified as hemoptysis, postinfectious bronchiectasis, radiological tree-in-bud score ≥2, and modified Reiff score ≥4 through multivariable analysis. The area under the receiver operating curve was 0.709 (95% confidence interval, .652–.766; *P* < .001).

NTM-LD­–associated factors were incorporated into an NTM-LD prediction model. The area under the receiver operating curve was 0.712 (95% CI, .655–.769; *P* < .001) (Figure 2). Based on the Youden index, the cutoff point was 2, indicating that ≥2 factors predicted NTM-LD with a sensitivity and specificity of 70.0% and 61.9%, respectively.

### NTM-LD Versus Non-NTM Outcomes

**Figure 3.**
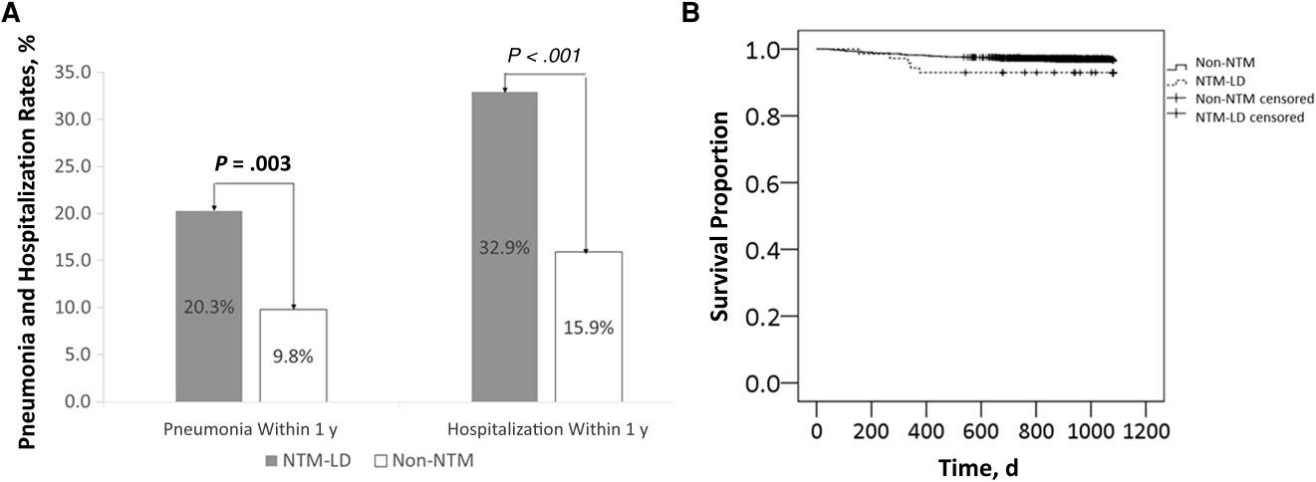
Outcomes of bronchiectasis comparing the nontuberculous mycobacteria (NTM) lung disease (NTM-LD) and non-NTM groups. *A*, Rates of pneumonia and hospitalization within 1 year. *B*, Kaplan-Meier 3-year survival curve comparing NTM-LD and non-NTM group. Patients in the NTM-LD group had significantly higher rates of pneumonia and hospitalization than those in the non-NTM group. The mortality rate at 3 years for individuals with NTM-LD was 6.3%, slightly higher than the 2.8% in those without NTM isolates.

Of the participants with NTM-LD, 20.3% (16 of 79) had pneumonia within 1 year of the index date, compared with 9.8% (248 of 2535) in the non-NTM group (*P* = .003) (Figure 3*A*). In the NTM-LD group, 32.9% (26 of 79) of the patients required hospitalization during the 1-year follow-up, which was significantly higher than in the non-NTM group (404 of 2535 [15.9%]; *P* < .001). Within 1 year, the mean numbers of admissions (SD) in the NTM-LD and non-NTM groups were 0.56 (1.10) and 0.27 (0.85), respectively (*P* = .003). The estimated 3-year mortality rate was slightly higher in the NTM-LD than in the non-NTM group (6.3% vs 2.8%; log-rank *P* = .086) (Figure 3*B*). The HR comparing 3-year mortality rates in NTM-LD and non-NTM groups was 2.09 (95% CI, .85–5.17; *P* = .11). In the year after the index date, 18 participants (22.8%) with NTM-LD underwent NTM-LD treatment. Compared with no anti-NTM treatment, NTM-LD treatment did not significantly affect hospitalization (mean number of admissions [SD], 0.67 [0.84] vs 0.52 [1.16], respectively; *P* = .63) and 3-year mortality (0% vs 9.3%; HR, 0.032 [95% CI, <.001 to 184.7]; *P* = .44] rates.

### Potential Pathogenic Microorganism Coisolates

**Figure 4.**
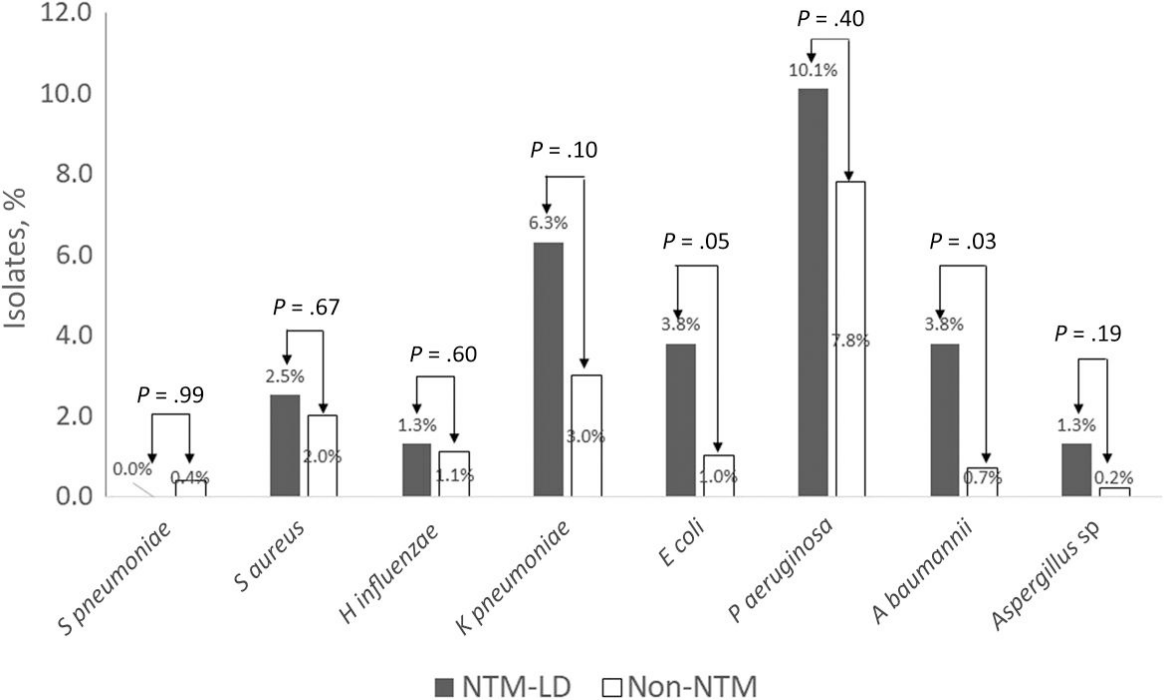
Coisolates of potential pathogenic microorganisms in individuals with nontuberculous mycobacteria (NTM) lung disease (LD) and those without any NTM isolation. *Pseudomonas aeruginosa*, *Klebsiella pneumoniae*, *Escherichia coli*, and *Acinetobacter baumannii* were the 4 most commonly coisolated bacteria in NTM-LD. There was no significant difference in the presence of *P aeruginosa* between NTM-LD and non-NTM groups, but *E coli* and *A baumannii* occurred more frequently in the NTM-LD group. Abbreviations: *H influenzae*, *Haemophilus influenzae*; *S aureus*, *Staphylococcus aureus*; *S pneumoniae, Streptococcus pneumoniae*.

Regardless of the group, *P aeruginosa* was the most common pathogenic microorganism (PPM) found in bronchiectasis (205 of 2614 [7.8%]), with similar *P aeruginosa* isolate rates in the NTM-LD and non-NTM groups (10.1% vs 7.8%; *P* = .40) (Figure 4). In the NTM-LD group, in the order of frequency, *P aeruginosa* was followed by *Klebsiella pneumoniae*, *Acinetobacter baumannii*, *Escherichia coli*, and *Staphylococcus aureus*. The non-NTM group had significantly fewer *A baumannii* and *E coli* isolates. In the NTM-LD group, the *K pneumoniae* isolate rate tended to be higher than in the non-NTM group (6.3% vs 3.0%; *P* = .10). However, *Streptococcus pneumoniae*, *Staphylococcus aureus*, *Haemophilus influenzae*, and *Aspergillus* species rates did not differ significantly between the groups.

## DISCUSSION

This study shows that patients with bronchiectasis and NTM-LD had a higher pneumonia risk than those with bronchiectasis without NTM-LD or colonization. Regardless of NTM-LD presence, *P aeruginosa* was the most common PPM coisolate. A US cohort study reported that, irrespective of NTM-LD status, hospitalization and pneumonia rates were similar, and that *P aeruginosa* incidence was only about 4% [19]. Despite controversial outcome reports, NTM-LD identification is essential, especially before beginning long-term macrolide therapy. The indicators assessed in this study, such as hemoptysis, postinfectious bronchiectasis (not COPD), bronchiectasis radiological severity, and TIB involvement might identify possible NTM-LD cases.

Timely and accurate NTM-LD diagnosis in patients with bronchiectasis can be challenging. Because the diseases have similar clinical manifestations, NTM-LD diagnosis requires strong suspicion and further investigation. In such situations, clinical characteristic–based predictive models may help identify NTM-LD in patients with bronchiectasis. Although several studies have reported NTM-LD risk factors in patients with bronchiectasis, such as sex, age, BMI, bronchiectasis severity, and postinfectious bronchiectasis [20–22], their results are not always consistent [20]. In addition to clinical parameters, some studies have investigated characteristic NTM-LD radiological findings in patients with bronchiectasis and suggested TIBs and extensive radiological involvement as risk factors [23, 24]. However, studies on NTM-LD predictive models in patients with bronchiectasis are scarce. To develop an NTM-LD risk model, the current study incorporated clinical and radiological factors and although the model’s predictive power, sensitivity, and specificity were modest, it may indicate possible NTM-LD in clinical practice for further evaluation.

Patients with COPD and other chronic airway diseases are vulnerable to NTM infection [25], and the NTM-LD risk might be double in patients with COPD and bronchiectasis [26]. Surprisingly, this study observed a lower COPD association with NTM-LD in patients with bronchiectasis. Some observational studies have reported that postmenopausal, nonsmoking women with low BMI are more susceptible to NTM infection, with predominant middle lobe involvement, partly indicating Lady Windermere syndrome [15, 27–30]. The prevalence of COPD is relatively low in such populations, suggesting that, in patients with bronchiectasis, NTM-LD might counteract the COPD risk. Therefore, regardless of NTM-LD presence, some studies did not find notable variation in COPD prevalence in patients with bronchiectasis [15, 27].

However, some studies reported a lower COPD prevalence in patients with bronchiectasis and NTM-LD [31], which is consistent with the current study's findings. The current study illustrated that NTM-LD more frequently involves the upper and middle lobes and not the lower lobes (Supplementary Figure 2). Modified Reiff scores indicated that the middle lobes were more severely involved in the NTM-LD than in the non-NTM group (Supplementary Figure 3). In consideration of BMI and smoking status, the subgroup of postmenopausal slender women without smoking, mainly involving middle lobes, was relatively predominant in NTM-LD in bronchiectasis in the study population, which might lower the risk of developing NTM-LD in COPD.

In bronchiectasis, PPM isolation from respiratory specimens is not uncommon, especially simultaneously with NTM-LD (17). With a prevalence of 27%–52%, *P aeruginosa* is the most frequently identified pathogen [32], and since it affects bronchiectasis prognosis significantly, the bronchiectasis severity index and FACED scores include *P aeruginosa* colonization as a severity evaluation factor [4, 5]. The current study shows that in patients with bronchiectasis, *P aeruginosa* was the most prevalent co-PPM in NTM-LD and non-NTM cases. Notably, compared with non-NTM cases, the odds of *P aeruginosa* isolation were not significantly higher in NTM-LD cases.

In addition, *H influenzae* accounted for only 1% of PPMs in patients with bronchiectasis in this study. However, compared with non-NTM cases, the risk of obtaining *E coli*, *K pneumoniae*, or *A baumannii* isolates was higher in NTM-LD. In Taiwan, *A baumannii* was reported as an important PPM in postinfectious bronchiectasis [10]. Because the current study indicates that NTM-LD might be associated with pneumonia, hospitalization, and increased mortality rate, proper and timely antibiotic use is crucial for pneumonia treatment [33]. In addition, our findings emphasize that, when choosing empirical antibiotics for pneumonia, it is important to consider the local drug sensitivities of PPMs like *P aeruginosa*, *A baumannii*, *E coli*, and *K pneumoniae*.

A meta-analysis of relevant studies published in 2006–2021 reported that NTM-LD prevalence in patients with bronchiectasis was 10%, with significant geographic variations [8], and US and Indian registry cohort studies reported frequencies of 30%–45% [13, 22]. However, a South Korean study reported a 5-year prevalence of only 4.5% [1]. Using medical records from branch hospitals of a single medical group to investigate NTM-LD prevalence in Taiwan from 2001 to 2016, Huang et al [10] reported a prevalence of 0.3% only. However, because of the exclusion from the study of patients with a prior tuberculosis history, who are at a higher risk of NTM-LD, the prevalence of NTM-LD may have been significantly underestimated. Unlike prevalence, the annual NTM-LD incidence data in patients with bronchiectasis is relatively scarce, probably because comprehensive mandatory reporting is lacking. In this study, the NTM-LD incidence was 29 cases per 1000 person-years, consistent with the incidence of 32.3 cases per 1000 person-years observed in a single-hospital cohort registry in Korea [31].

The current study has some limitations. First, because it is retrospective, the microbiological survey and timing were not strictly regulated, and NTM-LD incidence and PPM coisolation might therefore be underestimated. Second, PPM, NTM culture, pneumonia, and hospitalization dates were not obtained. Although this study found an NTM-LD association with pneumonia and hospitalization, a definite causal relationship could not be established. Furthermore, data were lacking on caustic pathogens in pneumonia and pneumonia treatment antibiotic choice. Therefore, a prospective registry is needed to validate these findings.

Third, because participants were enrolled in June 2020 with mortality data collected in June 2022, some participants might have been censored before the 3-year mark, potentially influencing the validity of the estimated 3-year mortality rate. Fourth, because of the lack of participants’ longitudinal comprehensive medical history from childhood to adulthood, it is difficult to differentiate postinfectious bronchiectasis from bronchiectasis with an infectious exacerbation. This might bias estimates of postinfectious bronchiectasis prevalence. Moreover, because the study mainly sought to obtain clinical data within 1 year of the index date, it could not provide information about NTM-LD development after the 1-year study period. In addition, because the recommended NTM-LD treatment duration is ≥12 months after sputum conversion [17], treatment responses could not be analyzed because treatment data beyond the study period were lacking. Finally, although MAC and MABC subspecies vary in virulence and drug sensitivity [34, 35], specific subspecies might not be always identified at study sites, and the lack of NTM subspecies data might bias the clinical impact of NTM-LD in patients with bronchiectasis.

In conclusion, the model proposed in this study has modest NTM-LD predictive power in patients with bronchiectasis. Nonetheless, ≥2 risk factors, such as hemoptysis, postinfectious bronchiectasis (not COPD), a modified Reiff score ≥4, and a TIB score ≥2 might indicate the possibility of simultaneous NTM-LD. In patients with bronchiectasis, NTM-LD was associated with pneumonia and hospitalization. In addition, compared with non-NTM cases, *P aeruginosa*, *E coli, A baumannii*, and *K pneumoniae* were more prevalent in NTM-LD cases. Therefore, there may be additional considerations about antibiotic choice for treating NTM-LD–exacerbated bronchiectasis.

## Supplementary Material

ofae427_Supplementary_Data

## Supplementary Data

Supplementary materials are available at *Open Forum Infectious Diseases* online. Consisting of data provided by the authors to benefit the reader, the posted materials are not copyedited and are the sole responsibility of the authors, so questions or comments should be addressed to the corresponding author.
