# Supplementary material for: Clinical Predictors of Nontuberculous Mycobacteria Lung Disease and Coisolates of Potential Pathogenic Microorganisms in Noncystic Fibrosis Bronchiectasis
Source: Open Forum Infect Dis. 2024 Jul 18;11(8):ofae427. doi: 10.1093/ofid/ofae427 (PMC11322833; doi:10.1093/ofid/ofae427)
Supplement: ofae427_Supplementary_Data [file ofae427_supplementary_data.docx]

Table S1 The species distribution of non-tuberculous mycobacteria colonization in bronchiectasis

| MAC | MABC | *M. fortuitum* | *M. gordonae* | *M. kansasii* | *M. xenopi* | *M. chelonae* | NTM | Mixed |
| --- | --- | --- | --- | --- | --- | --- | --- | --- |
| 28 (27.2) | 17 (16.5) | 13 (12.6) | 12 (11.7) | 7 (6.8) | 4 (3.9) | 3 (2.9) | 9 (8.7) | 10(9.7)* |

Data presented as number (%)

* Two MABC/MAC, one unspecific NTM/MAC, one NTM/M. fortuitum, one NTM/M. gordonae, one NTM/MK one M fortuitum/M. gordonae, one MABC/M. xenopi, one MAC/MK, and one MAC/M. gordonae

Abbreviation: MABC: *Mycobacterium abscessus complex*; MAC: *Mycobacterium avium complex*; NTM: unspecific non-tuberculous mycobacteria

Table S2 The data of pulmonary function test

| Pulmonary function test | Total  (n = 1403) | Non-NTM  (n = 1370) | NTM-LD  (n = 33) | p |
| --- | --- | --- | --- | --- |
| FEV_1_ (L/sec) | 1.55 ± 0.66 | 1.55 ± 0.66 | 1.51 ± 0.66 | 0.71 |
| FEV_1_ % predicted | 70.2 ± 24.8 | 70.3 ± 24.7 | 65.7 ± 29.3 | 0.30 |
| FVC (Liter) | 2.13 ± 0.84 | 2.14 ± 0.84 | 2.00 ± 0.80 | 0.36 |
| FVC% predicted | 76.2 ± 25.9 | 76.4 ± 25.9 | 70.1 ± 27.0 | 0.17 |
| FEV_1_/FVC < 0.7 | 484 (34.5) | 474 (34.5) | 10 (30.3) | 0.713 |

Abbreviations: FEV1: forced expiratory volume in one second; FVC: forced vital capacity; NTM-LD: nontuberculous mycobacteria-lung disease

Table S3 The status of macrolide use more than 90 days in non-NTM and NTM groups

|  | Non-NTM  (n = 2535) | NTM-LD  (n = 79) | p |
| --- | --- | --- | --- |
| Macrolide | 273 (10.8) * | 10 (12.7)† | 0.21 |

Data were presented as number (percent).

*: azithromycin: 56; erythromycin: 101; clarithromycin:120 (sequential use of two macrolides: 4)

†: azithromycin: 1; erythromycin: 3; clarithromycin:6

Macrolide use for anti-NTM treatment were excluded.

Abbreviations: NTM-LD: nontuberculous mycobacteria-lung disease

Figure S1. Flow chart of subjects’ enrollment


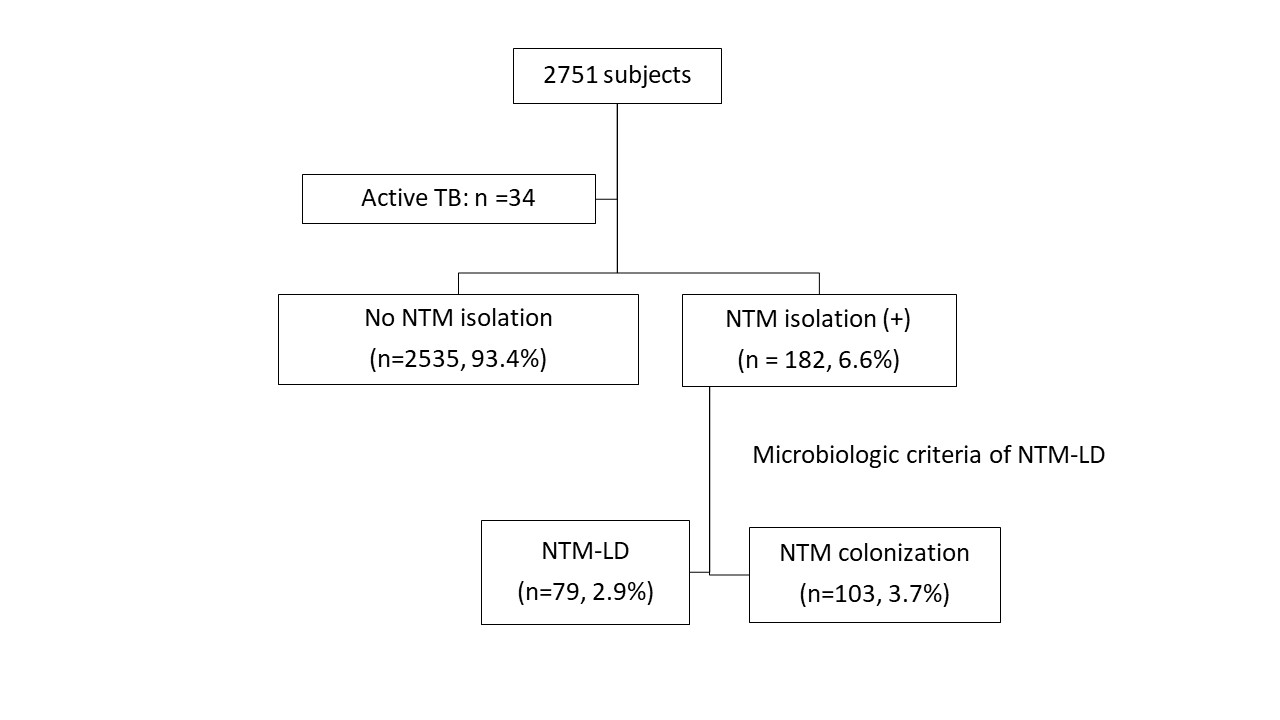
Abbreviations: NTM-LD: nontuberculous mycobacteria- lung disease; TB: tuberculosis

Figure S2. The proportion of bronchiectasis involvement in various lung lobes


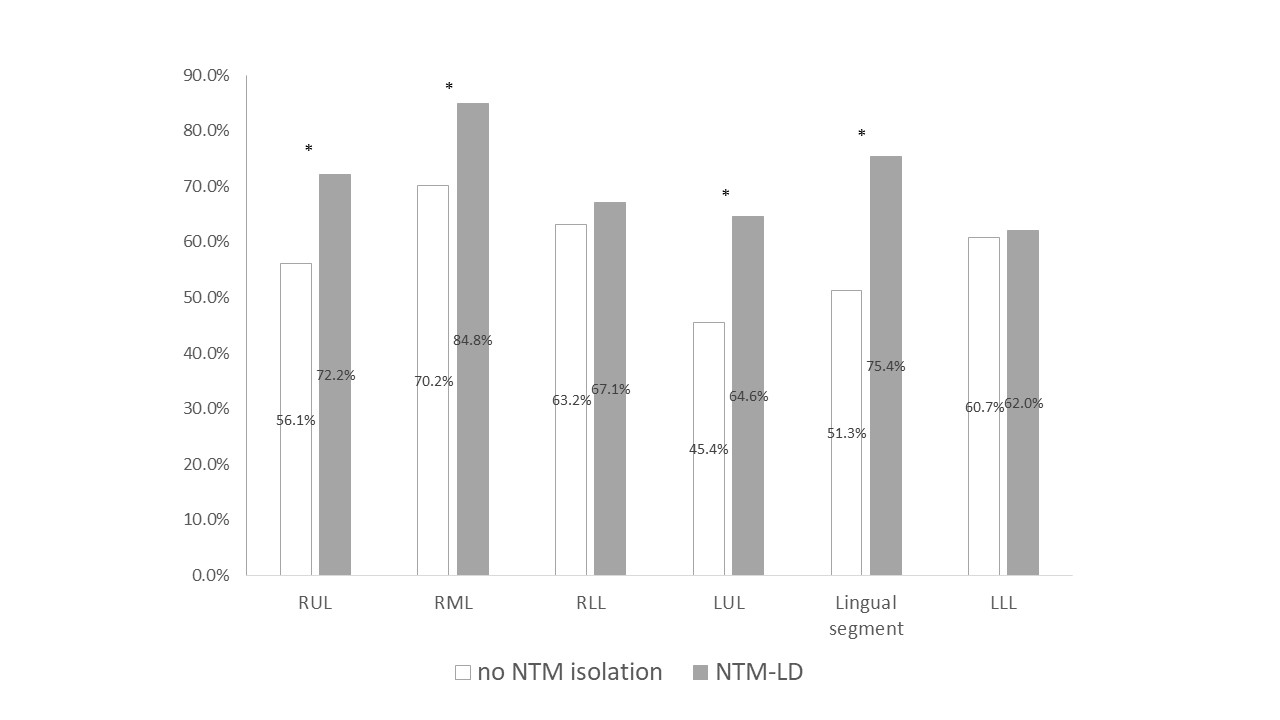


The proportion of bronchiectatic involvement of bilateral upper and middle lobes was more in NTM-LD than non-NTM

*: p < 0.05

Abbreviations: RUL: right upper lobe; RML: right middle lobe; RLL: right lower lobe; LUL: left upper lobe; LLL: left lower lobe; NTM-LD: nontuberculous mycobacteria- lung disease

Figure S3. Radiologic scores of bilateral upper and middle lobes, NTM-LD vs. non-NTM


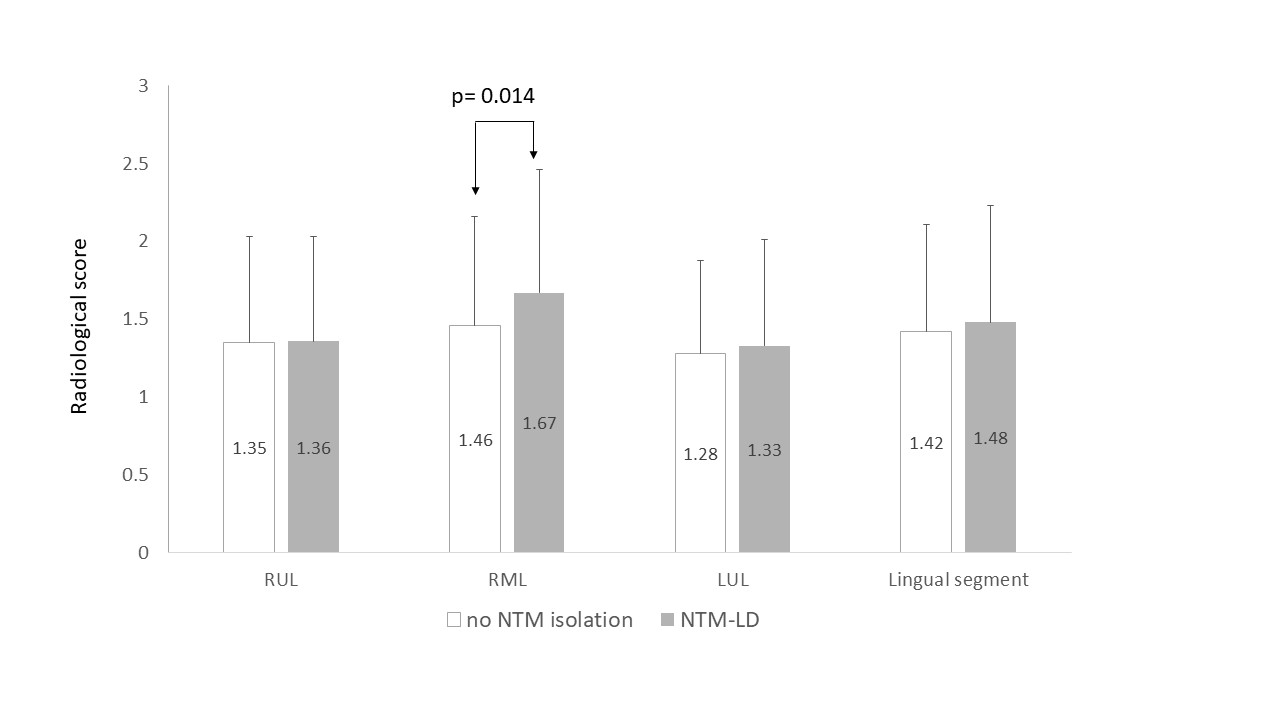


The radiologic modified Reiff score of RML was more severe in NTM-LD than non NTM.

Abbreviations: RUL: right upper lobe; RML: right middle lobe; RLL: right lower lobe; LUL: left upper lobe; NTM-LD: nontuberculous mycobacteria- lung disease
